# Lovastatin Inhibits EMT and Metastasis of Triple-Negative Breast Cancer Stem Cells Through Dysregulation of Cytoskeleton-Associated Proteins

**DOI:** 10.3389/fonc.2021.656687

**Published:** 2021-06-04

**Authors:** Chanjuan Zheng, Shichao Yan, Lu Lu, Hui Yao, Guangchun He, Sisi Chen, Ying Li, Xiaojun Peng, Zhongyi Cheng, Mi Wu, Qiuting Zhang, Guifei Li, Shujun Fu, Xiyun Deng

**Affiliations:** ^1^ Key Laboratory of Model Animals and Stem Cell Biology in Hunan Province, Departments of Pathology and Pathophysiology, Hunan Normal University School of Medicine, Changsha, China; ^2^ Key Laboratory of Translational Cancer Stem Cell Research, Hunan Normal University, Changsha, China; ^3^ Department of Preventive Medicine, Hunan Normal University School of Medicine, Changsha, China; ^4^ Jingjie PTM BioLab Co. Ltd., Hangzhou, China

**Keywords:** lovastatin, triple-negative breast cancer, cancer stem cells, epithelial-to-mesenchymal transition, cytoskeleton

## Abstract

Triple-negative breast cancer (TNBC) is more aggressive and has poorer prognosis compared to other subtypes of breast cancer. Epithelial-to-mesenchymal transition (EMT) is a process in which epithelial cells transform into mesenchymal-like cells capable of migration, invasion, and metastasis. Recently, we have demonstrated that lovastatin, a 3-hydroxy-3-methylglutaryl-coenzyme A reductase inhibitor and a lipid-lowering drug, could inhibit stemness properties of cancer stem cells (CSCs) derived from TNBC cell *in vitro* and *in vivo*. This study is aimed at investigating whether lovastatin inhibits TNBC CSCs by inhibiting EMT and suppressing metastasis and the mechanism involved. In the present study, we found that lovastatin dysregulated lysine succinylation of cytoskeleton-associated proteins in CSCs derived from TNBC MDA-MB-231 cell. Lovastatin inhibited EMT as demonstrated by down-regulation of the protein levels of Vimentin and Twist in MDA-MB-231 CSCs *in vitro* and *vivo* and by reversal of TGF-β1-induced morphological change in MCF10A cells. Lovastatin also inhibited the migration of MDA-MB-231 CSCs. The disruption of cytoskeleton in TNBC CSCs by lovastatin was demonstrated by the reduction of the number of pseudopodia and the relocation of F-actin cytoskeleton. Combination of lovastatin with doxorubicin synergistically inhibited liver metastasis of MDA-MB-231 CSCs. Bioinformatics analysis revealed that higher expression levels of cytoskeleton-associated genes were characteristic of TNBC and predicted survival outcomes in breast cancer patients. These data suggested that lovastatin could inhibit the EMT and metastasis of TNBC CSCs *in vitro* and *in vivo* through dysregulation of cytoskeleton-associated proteins.

## Introduction

Breast cancer is by far the most common malignancy and the second leading cause of cancer-related mortality among women (1). It is a type of heterogeneous disease that differs in pathomorphology, biology, clinical manifestation, and treatment response (2). Triple-negative breast cancer (TNBC), as a subtype of breast cancer, accounts for 10-20% of them. Owing to the lack of expression of estrogen receptor (ER), progesterone receptor (PR) and lack of expression or amplification of human epidermal growth factor receptor 2 (HER2), TNBC patients are insensitive to endocrine therapy or molecular targeted therapy, resulting in high recurrence and metastatic potential (3). Currently, new treatment strategies for TNBC, including poly (ADP-ribose) polymerase (PARP) inhibition and immune checkpoint inhibition, are being actively developed in preclinical and clinical studies (4, 5). Unfortunately, only a small proportion of TNBC patients could benefit from these treatments (6, 7). Therefore, it is an urgent task to explore promising targeted drugs so as to improve the efficacy for TNBC.

There is substantial evidence that breast cancer development is hierarchically organized and driven by a minute population of cancer cells known as cancer stem cells (CSCs) which contribute to tumor metastasis and relapse (8, 9). Targeting CSCs has become a popular goal for treating a wide range of tumor types, and may be especially important for TNBC patients (10). Numerous clinical trials have been conducted for targeting breast cancer CSCs (11), but limited data currently exist clinically for the treatment of TNBC (10). Therefore, discovery of drugs that target CSCs will have an enormous impact in TNBC therapeutics. In the process of cancer metastasis, CSCs undergo epithelial-to-mesenchymal transition (EMT), thereby acquiring mesenchymal features which have the ability to migrate and invade (12, 13). EMT involves the loss of intracellular cohesion, disruption of the extracellular matrix (ECM), modifications of the cytoskeleton, and increased cell motility and invasiveness (14, 15). Accumulating evidence showed that EMT-inducible factors also enhance or induce CSC-like features in cancer cells. Within breast cancer, the acquisition of tumor stem cell-like features, the formation of tumor spheres and the appearance of a breast cancer stem cell-specific phenotype (CD44^+^/CD24^-^) were all promoted by the occurrence of EMT (16).

Post translational modifications (PTMs) are one of the most efficient biological mechanisms for expanding the genetic code and for regulating cellular pathophysiology. Lysine succinylation (Ksucc), a newly identified form of PTMs, could cause significant changes in the structure and function of proteins (17). Several lines of evidence suggest that Ksucc has been involved in the initiation and development of numerous different types of tumors, such as gastric and breast cancer (18–20). However, Ksucc also exerts tumor-inhibitory effect in hepatocellular carcinoma and intestinal cancer (21, 22). Importantly, the mechanism of some anti-tumor drugs may also be related to Ksucc modification. For example, heat shock protein 90 (HSP90) inhibitor exhibits an anti-tumor activity against bladder cancer by affecting Ksucc modification (23).

Lovastatin is a natural statin derived from *Monascus*-fermented rice or dioscorea and occurs at a high content in *Oyster* mushroom (24). It has been widely used in prevention and treatment of hyperlipidemia (25). In the last two decades, the antitumor effect of lovastatin has gained increasing attention (26). *In vitro* studies have shown that lovastatin could inhibit the cell cycle progression (27), induce apoptosis (28), and suppress cell migration and invasion (29). *In vivo*, lovastatin could suppress the growth of transplanted tumor or prevent pulmonary metastasis derived from breast cancer (27). Recently, we have demonstrated that lovastatin could inhibit stemness properties of CSCs derived from TNBC cell lines *in vitro*, in a mouse model of orthotopic tumor growth, and in a patient-derived xenograft (PDX) model ( (30–32) and manuscript in preparation). Our findings support the evidence that lovastatin may be a candidate drug for the treatment of TNBC.

Through global profiling of lysine acylation, we found that lovastatin preferentially targets CSCs derived from TNBC over non-TNBC cells through Ksucc of proteins involved in cytoskeleton. Our studies demonstrated that lovastatin could selectively inhibit the viability of TNBC CSCs *in vitro* and *in vivo*. Therefore, this study aims to further investigate whether lovastatin exerts its anticancer effect in TNBC CSCs through inhibiting the EMT program and metastasis *via* regulation of cytoskeleton-associated proteins.

## Materials and Methods

### Key Reagents

Lovastatin (ab120614) was obtained from Abcam (Cambridge, UK) and dissolved in DMSO at a stock concentration of 20 or 30 mM and stored at -80°C before use. Doxorubicin was purchased from Selleck, dissolved in DMSO and stored as directed. Human recombinant TGF-β1 was purchased from R&D Systems (Minneapolis, MN) and was dissolved in an aqueous solvent (vehicle) containing 4 mM HCl and 1 mg/ml BSA.

### Cell Lines

Breast cancer cell lines MDA-MB-231 (TNBC) and MDA-MB-453 (non-TNBC) were purchased from the Cell Resource Center of Shanghai Institutes for Biological Sciences, maintained in DMEM medium supplemented with 10% fetal bovine serum. The immortalized mammary epithelial cell line MCF10A was obtained from Kunming Institute of Zoology, Chinese Academy of Sciences. MCF10A cells were maintained in DMEM/F-12 medium supplemented with 5% horse serum, 20 ng/mL EGF, 500 ng/mL hydrocortisone, 100 ng/mL cholera toxin and 10 μg/L insulin. All cell lines were routinely cultured at 37°C with 21% O_2_ and 5% CO_2_ and were tested negative for mycoplasma contamination.

### Enrichment and Characterization of Breast Cancer Stem Cells

MDA-MB-231 or MDA-MB-453 cells were trypsinized to single cells and seeded into 6-well ultra-low attachment plates (2,500 cells/mL) using breast cancer stem cell medium (DMEM/F12, 1× B27, 20 ng/mL EGF, 20 ng/mL bFGF, 0.4% BSA, 4 μg/mL insulin and 0.2% hydrocortisone) (33). CD44^+^/CD24^-^ cells were sorted by sequential magnetic sorting after addition of beads coated with anti-CD44 or anti-CD24 antibody according to our published protocol (30). These cells with CSC-like properties were designated sphere-forming cells (SFCs) to distinguish from their parental cells (PCs). The CSC phenotype was characterized by prolonged mammosphere formation in ultra-low attachment culture and by their enhanced tumorigenic ability as demonstrated by two orders of magnitude higher tumorigenicity of SFCs than PCs in nude mice (**Supplementary Figure 1** and **Supplementary Table 1**).

### Immunofluorescence – Laser Scanning Confocal Microscopy

The cover glasses were put into 6-cm dishes, and MDA-MB-231 CSCs (1 × 10^5^ cells/mL) were seeded and allowed to grow overnight at 37°C. The cells were treated with different concentrations of lovastatin for 48 hours. These cover glasses were fixed with 4% paraformaldehyde and collected for indirect immunofluorescence staining. The primary antibodies included those against Vimentin (ZSGB-BIO, Cat#ZM0260, Mouse, 1:100) or Twist (Abcam, Cat#ab50581, Rabbit, 1:100). The secondary antibodies were DyLight 488 anti-mouse IgG (H+L) (Vector, Cat#DI-2488, 1:100) and DyLight 594 anti-rabbit IgG (H+L) (Vector, Cat#DI-1594, 1:100), respectively.

### Western Blot Analysis

Cultured cells were lysed using 1× cell lysis buffer (Cell Signaling Technology, Danvers, MA, USA) with 1× protease inhibitor cocktail (Complete Mini, Roche, Mannheim, Germany) and 1 mM phenylmethanesulfonyl fluoride (Sigma-Aldrich) added. After centrifugation, the supernatants (whole cell lysates) were collected and quantified by the BCA protein quantification method. The lysates were mixed with the LDS sample buffer and reducing agent and denatured by boiling. The same quantity of protein from each sample was then separated on 10% denaturing PAGE gels followed by incubation with the respective primary antibodies (Vimentin, CST, Cat#5741, 1:1,000; Twist, Abcam, Cat#ab50581, 1:1,000; GAPDH, ZSGB-BIO, Cat#TA08, 1:10,000) and the HRP-conjugated secondary antibody followed by subsequent ECL development according to our standard procedure (34).

### Nude Mouse Models

Balb/c-nu mice (female, 5 – 6 weeks old, weight 16 – 18 g) were purchased from Hunan SJA Laboratory Animals Co., Ltd. The mice were maintained on a regular sterile diet under SPF animal house conditions. For the model of orthotopic tumor growth and EMT phenotype (**Figure 3A**), CSCs resuspended in cold 1× PBS were injected into the fourth mammary fat pad. Two weeks later, the nude mice were randomly grouped based on tumor sizes (*n* = 10/group). Lovastatin (2 mg/kg) or vehicle (PBS) was administered twice weekly through oral gavage until the end of the experiment. The tumor growth was monitored by measuring the major (*a*) and minor (*b*) axes of the tumor using a caliper twice weekly. The tumor volume (*V*) was estimated by the equation *V* = (*a* × *b* ^2^)/2 as described (35). Three weeks after drug treatment, the mice were sacrificed and the tumors were resected, weighed, and photographed. Part of the tumor tissue was fixed in 4% buffered formaldehyde and subjected to routine paraffin-embedding and microtome sectioning.

A model of tumor metastasis (**Figure 6A**) was generated by injecting the CSCs (5 × 10^3^/100 μL/animal) into the tail vein of the nude mice. The mice were randomized into the following four groups (*n* = 8/group): saline control, doxorubicin (1 mg/kg), lovastatin (2 mg/kg), and doxorubicin (1 mg/kg) + lovastatin (2 mg/kg). Drug administration started the next day after tumor cell injection and continued twice weekly for 7 weeks. At the end of drug treatment, the mice were sacrificed, and the tumors and the livers resected, weighed, and photographed. Part of the tumor tissue was fixed in 4% buffered formaldehyde and subjected to routine paraffin-embedding, microtome sectioning, and H&E or immunohistochemical staining. Metastatic burden was evaluated by counting the metastatic nodules on the surface of each liver. Micrometastasis in the liver tissues were quantified based on the literature (36). All animal studies were approved by the Hunan Normal University Animal Care Committee.

### Immunohistochemistry

Immunohistochemical staining was carried out using the PV-9000 plus poly-HRP anti-mouse/rabbit IgG detection system as described in our previous study (37).The details of primary antibodies were as follows: Vimentin, CST, Cat#5741, 1:100; Twist, Abcam, Cat#ab50581, 1:100. After immunohistochemical staining, the tissue sections were scanned using Automated Quantitative Pathology Imaging System (Vectra, PerkinElmer, Hopkinton, MA, USA) and the total intensity score (TIS) was calculated each from six randomly chosen images at 40× magnification.

### Wounding Healing Assay

MDA-MB-231 or MDA-MB-453 CSCs were seeded at a density of 2 × 10^5^ cells/well in 6-well plates. When the cells have grown and fused to 80%, the tip head was used to scratch the central area of the plate well. Lovastatin (1 μM) was added to the cells cultured in DMEM medium supplemented with 3% fetal bovine serum. The cell migration distance in the scratch area was measured at 0 and 24 hours, respectively.

### LC-MS/MS Analysis and Data Search

The CSCs were treated with lovastatin (1 μM) or vehicle in stem cell medium at 37°C for 48 h. The cells were then collected by centrifugation, washed with PBS, and snap-frozen in liquid nitrogen, followed by protein extraction and trypsin digestion. The resulting peptides were labeled with tandem mass tag (TMT) isobaric reagents and fractionated by strong cation exchange chromatography. Succinylated peptides were immunoprecipitated with pan-Ksucc antibody-conjugated beads. Enriched peptides were analyzed by liquid chromatography coupled to an Orbitrap Q Exactive™ Plus. Non-enriched peptides (for proteomics) were fractionated by high pH reverse-phase HPLC using the Agilent 300 Extend C18 column followed by LC-MS/MS analysis. The resulting MS/MS data was processed using MaxQuant with integrated Andromeda search engine (v.1.5.2.8). Tandem mass spectra were searched against Swissprot human database concatenated with reverse decoy database. False discovery rate (FDR) thresholds for protein, peptide, and modification site were specified at 1%. Minimum peptide length was set at 7. For quantification method, TMT 6-plex was selected. The site localization probability was set at ≥ 0.75. The relative changes of Ksucc-modified proteins were normalized to the respective protein level revealed by global proteomic profiling.

### Bioinformatics Analyses of Gene Expression Levels and Breast Cancer Patient Survival

To explore the expression levels of cytoskeleton-related genes in TNBC and non-TNBC, we analyzed the RNA-seq data of 115 TNBC and 982 non-TNBC clinical samples from the cBioPortal database (http://www.cbioportal.org/study/summary?id=brca_tcga). After obtaining the results, we plotted the value of the ordinate to the gene expression converted by log10. We then integrated four datasets, i.e., GSE42568, Nathan Kline Institute (NKI), and GSE3494-U133A, and GSE1456-GPL97 to analyze the overall survival (OS) of breast cancer patients of all molecular subtypes between high and low expression levels of cytoskeleton-related genes. Results were obtained with the PROGgeneV2 tool (http://genomics.jefferson.edu/proggene/). We next explored the relationship between these cytoskeleton-related genes and the TNBC patients’ survival by Kaplan-Meier plotter database (http://kmplot.com/analysis/index.php?p=service&cancer=breast).

### Transmission Electron Microscopy

To investigate the formation of pseudopodia, the CSCs were treated with lovastatin (1 μM) or vehicle for 48 h, fixed with 2.5% glutaraldehyde, and post-fixed in 1% osmium tetroxide (OsO4) for 1 – 2 h at 4°C. The samples were then dehydrated in a graded series of acetone (50%, 70%, 90%, and 100%) and embedded in Epon-Araldite resin. Ultra-thin sections (50 – 100 nm) were cut using the ultramicrotome and stained with 3% uranyl acetate and lead nitrate. The cell morphology was observed, and the images acquired using an HT7700 Transmission Electron Microscope (Hitachi, Tokyo, Japan).

### Statistical Analysis

All the quantitative data were presented as mean ± SEM. Statistical analyses (ANOVA, unpaired Student’s *t* test) were carried out using SigmaPlot (version 12.5). *P* < 0.05 was considered as statistically significant. IC_50_ was calculated using the GraphPad Prism 5 software. Drug interaction between lovastatin and doxorubicin was assessed using the CompuSyn software to calculate the combination index (*CI*), with *CI <*1, *CI* = 1, and *CI >*1 indicating synergistic, additive, and antagonistic actions, respectively.

## Results

### Lovastatin Dysregulates Lysine Succinylation of Cytoskeleton-Associated Proteins

Lysine acylations, novel forms of post-translational modifications, play a key role in drug-induced cytotoxicity (38). To investigate the pathways of targeted by lovastatin in TNBC CSCs, we compared lysine acylations of TNBC cells (MDA-MB-231 CSCs) with non-TNBC cells (MDA-MB-453 CSCs). We found that lysine succinylation (Ksucc) was a major lysine acylation type dysregulated by lovastatin in MDA-MB-231 CSCs compared with MDA-MB-453 CSCs (data not shown).

**Figure 1:**
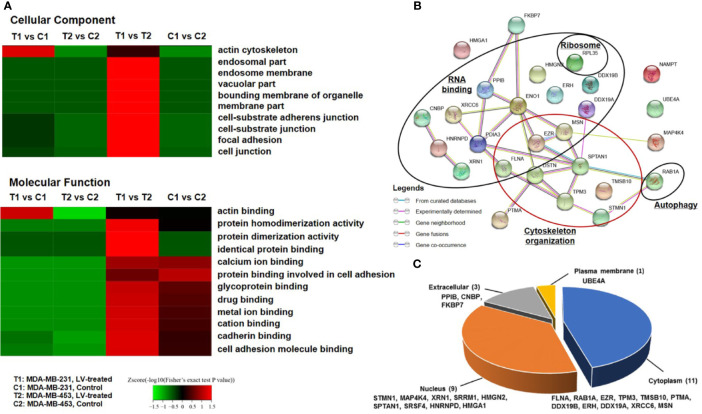
Lovastatin induces lysine succinylation (Ksucc) of cytoskeleton-associated proteins. Gene Ontology (GO) analysis showing the enrichment of Ksucc-modified proteins involved in cytoskeleton organization in MDA-MB-231 CSCs treated with lovastatin (1 μM, 48 h) **(A)**. Protein-protein interaction network generated using the STRING database (https://string-db.org/) showing the functional groups of lovastatin-dysregulated Ksucc-modified proteins in MDA-MB-231 CSCs **(B)**. Pie-chart showing the distribution of Ksucc-modified proteins in different cellular components of MDA-MB-231 CSCs **(C)**. LV, lovastatin.

We next performed TMT labeling and immunoprecipitation using pan-Ksucc antibody followed by LC-MS/MS to uncover the changes of Ksucc modifications and the specific sites. Bioinformatics analyses were performed to annotate the proteins differentially modified by Ksucc in response to lovastatin treatment. Gene Ontology (GO) analysis showed that Ksucc-modified proteins were mainly involved in cytoskeleton organization (such as actin binding) (**Figure 1A**). Protein-protein interaction network analysis based on Search Tool for the Retrieval of Interacting Genes (STRING) (http://string-db.org/) showed the proteins (FLNA, TMSB10, STMN, TPM3, MSN, SPTAN1, DSTN, and EZR) of cytoskeleton organization as key mediators of lovastatin’s action in MDA-MB-231 CSCs (**Figure 1B**). Subcellular localization analysis revealed that most of the succinylated proteins were distributed in the cytoplasm and the nucleus (**Figure 1C**), consistent with their localization and role in regulating the cytoskeleton organization.

### Lovastatin Inhibits Epithelial-to-Mesenchymal Transition of TNBC CSCs

**Figure 2:**
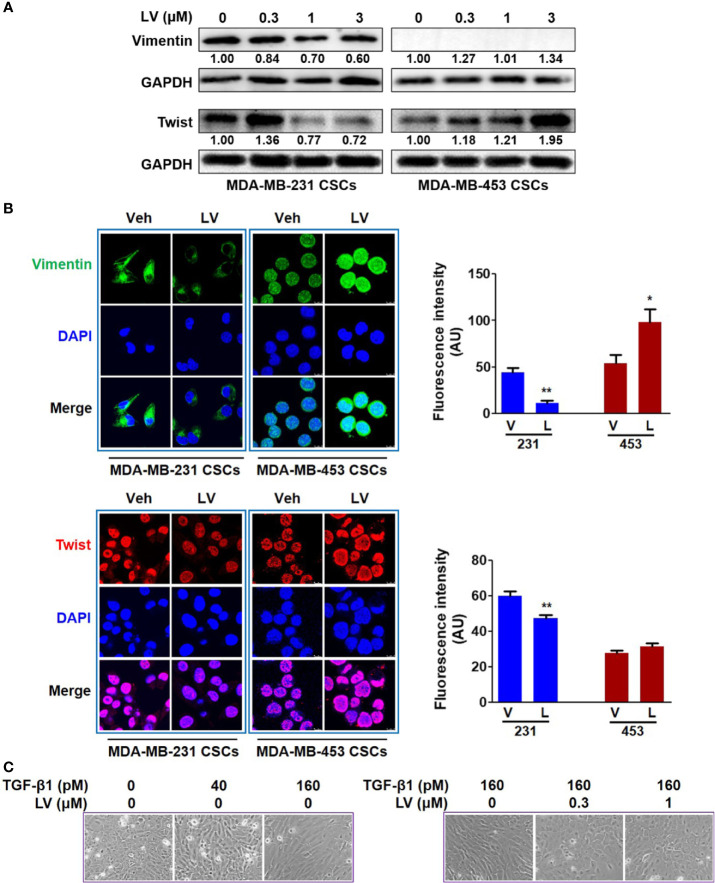
Lovastatin inhibits EMT of TNBC CSCs *in vitro*. MDA-MB-231 CSCs and MDA-MB-453 CSCs were treated with lovastatin or vehicle for 48 h and the protein levels of Vimentin and Twist were analyzed by western blot analysis **(A)**. Representative confocal images immunofluorescence staining of Vimentin (green) and Twist (red) on MDA-MB-231 CSCs treated in a similar way as in **(A)**. Nuclei were stained with 4′, 6-diamidino-2-phenylindole (DAPI) (blue). Original magnification: 63×. Right, quantification of immunofluorescence intensity **(B)**. MCF10A cells were cultured for 7 days with or without different concentrations of TGF-β1 and/or lovastatin, morphological changes were observed by microscopic examination. Original magnification: 200× **(C)**. *P < 0.05, **P < 0.01, compared with control; V or Veh, vehicle; L or LV, lovastatin; AU, arbitrary unit.

MDA-MB-231 and MDA-MB-453 CSCs were treated with different concentrations of lovastatin (0.3 – 3 μM) for 48 h. Western blot analysis revealed that the protein levels of Vimentin and Twist were decreased by lovastatin treatment in MDA-MB-231 but not MDA-MB-453 CSCs (**Figure 2A**). We noticed the increased protein level of Twist in MDA-MB-231 CSCs treated with 0.3 μM lovastatin. This may suggest a dose-dependent effect for lovastatin on some of its actions, which was consistent with other results obtained with this drug in our hands (unpublished observations). The expression of Vimentin and Twist was investigated by immunofluorescence and laser scanning confocal microscopy. The fluorescence intensity of Vimentin (green) and Twist (red) was decreased by lovastatin treatment in MDA-MB-231 but not MDA-MB-453 CSCs (**Figure 2B**).

The effect of lovastatin on EMT was further investigated by another cell model of induced EMT. Addition of TGF-β1 to immortalized epithelial cells such as MCF10A is a well-recognized cell model of inducing EMT *in vitro* (39). When treated with TGF-β1 (160 pM), MCF10A cells could form the loose linked spindle morphology. Addition of lovastatin (0.3 – 1 μM) reversed the change of cell morphology induced by TGF-β1 (**Figure 2C**). These results demonstrate the inhibitory effect of lovastatin on the EMT of TNBC CSCs.

**Figure 3:**
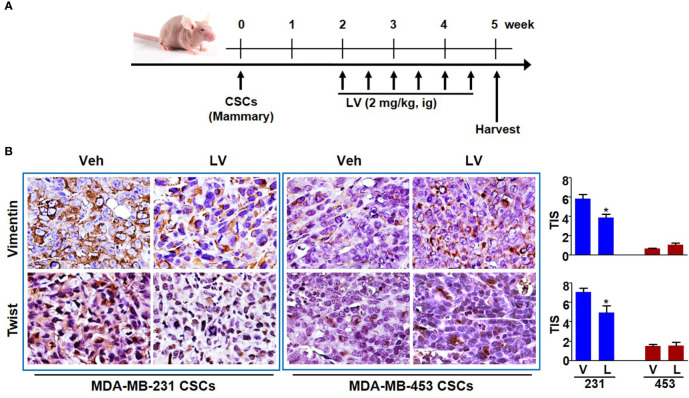
Lovastatin inhibits EMT of TNBC CSCs *in vivo*. Schematic diagram showing the experimental procedure of mouse model of EMT phenotype **(A)**. Representative images of immunohistochemical staining for Vimentin and Twist in orthotopic tumors derived from MDA-MB-231 and MDA-MB-453 CSCs. The nucleus was counterstained by hematoxylin. Right, quantification of the total intensity score (TIS) **(B)**. *P < 0.05, compared with control; V or Veh, vehicle; L or LV, lovastatin; ig, intragastric administration.

The orthotopic xenograft model of mammary fat pad injection was used to study the tumor growth and EMT phenotype (**Figure 3A**). Each of the tumors was spherical or irregular in shape and gray or gray-red in color. For the mice receiving MDA-MB-231 CSCs, the average tumor volume of the lovastatin-treated group was smaller than that of the control group (*P* < 0.05) (**Supplementary Figure 2A** **)**. For the mice receiving MDA-MB-453 CSCs, the average tumor volume of the lovastatin-treated group was even larger compared with the control group (*P* < 0.05). Tumor weight analysis at the end of the experiment confirmed the results of tumor volume measurement (**Supplementary Figure 2B**). Immunohistochemical staining was performed to evaluate the EMT-related proteins on the orthotopic tumors. We found that in xenograft tumors derived from MDA-MB-231 CSCs, the lovastatin-treated group had a lower score of the mesenchymal markers Vimentin and Twist than the control group (*P* < 0.05) (**Figure 3B**). Again, in MDA-MB-453 CSCs tumors, there was no statistical difference in Vimentin and Twist between the lovastatin-treated group and the control group.

### Lovastatin Promotes Chemosensitization and Inhibits Metastasis of TNBC CSCs

**Figure 4:**
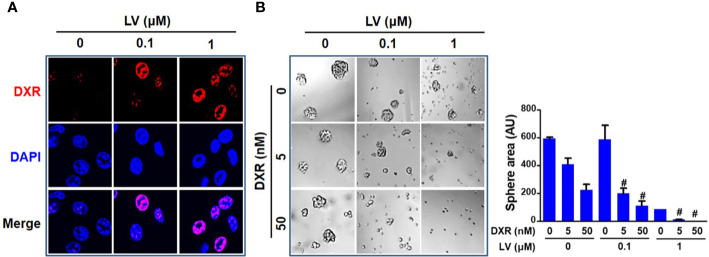
Lovastatin increases the sensitivity of TNBC CSCs to doxorubicin. Intracellular accumulation of DXR promoted by lovastatin. MDA-MB-231 CSCs were treated for 24 h with DXR (5 μM), alone or in combination with lovastatin, and the intracellular fluorescence of DXR (red) was observed by laser scanning confocal microscopy. Original magnification: 63× **(A)**. Synergistic effect between lovastatin and DXR on inhibiting tumorsphere-forming activity. MDA-MB-231 CSCs were cultured in the presence or absence of lovastatin and/or DXR and the tumorspheres were observed and recorded 5 d after treatment. Right, quantifications of the areas of tumorspheres **(B)**. ^#^ *CI* < 1.0, showing synergism between the two drugs. LV, lovastatin; DXR, doxorubicin; AU, arbitrary unit.

Since CSCs contribute to chemoresistance (40), we next investigated whether lovastatin synergizes with the standard chemotherapeutic drug to elicit greater inhibitory effect. We demonstrated that lovastatin sensitizes MDA-MB-231 CSCs to doxorubicin, a standard chemotherapeutic drug for breast cancer therapy. Confocal microscopy of autofluorescence revealed that lovastatin promoted intracellular accumulation of doxorubicin in MDA-MB-231 CSCs (**Figure 4A**). Furthermore, lovastatin synergized with doxorubicin to inhibit tumorsphere formation of MDA-MB-231 CSCs (**Figure 4B**).

**Figure 5:**
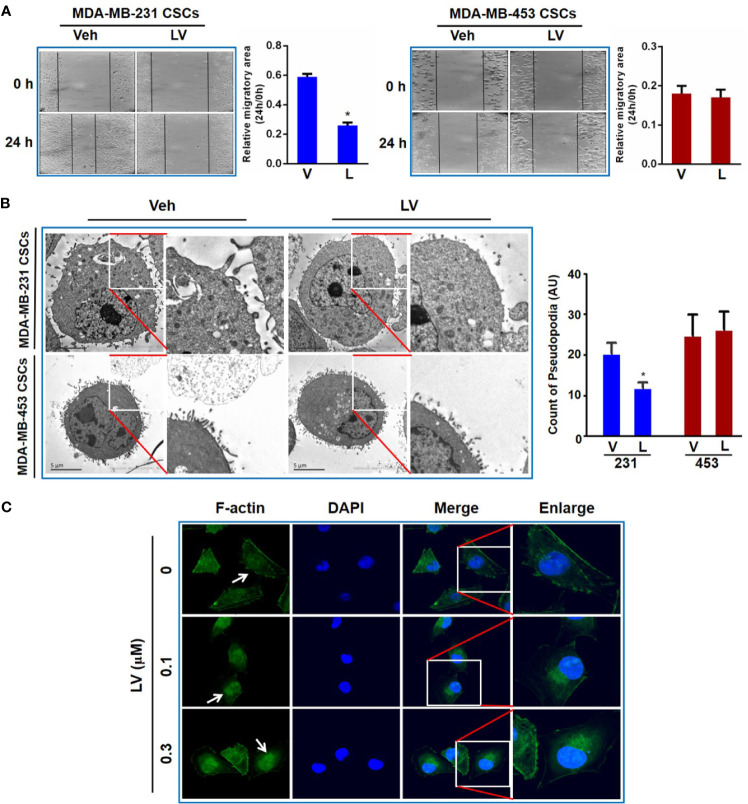
Lovastatin inhibits migration of TNBC CSCs *in vitro*. Representative cell images of wound-healing assay in MDA-MB-231 and MDA-MB-453 CSCs treated with vehicle or lovastatin (1 μM, 24 h). Right, quantification of relative migratory area **(A)**. Representative TEM micrographs showing the pseudopodia in MDA-MB-231 and MDA-MB-453 CSCs after treatment with lovastatin (1 μM, 48 h). Scale bar = 5 μm **(B)**. Representative confocal images of immunofluorescence staining for F-actin in MDA-MB-231 after treatment with vehicle or lovastatin (0.1 or 0.3 μM, 48 h). Blue, DAPI staining of the nucleus. Original magnification: 63× **(C)**. *P < 0.05, compared with control; V or Veh, vehicle; L or LV, lovastatin; AU, arbitrary unit.

Wounding healing assay was used to evaluate the effect of lovastatin on TNBC CSCs migration. CSCs were treated with lovastatin and photographed at 0 and 24 h respectively after cell scratching. We found that the migration area of the lovastatin-treated group was significantly larger than that of the vehicle-treated group in MDA-MB-231 CSCs. However, there was no obvious inhibitory effect on migration in MDA-MB-453 CSCs (**Figure 5A**).

Considering the formation of pseudopodia is supported by actin cytoskeleton, we evaluated whether lovastatin caused disruption of pseudopodia in TNBC cells. As expected, transmission electron microscopy (TEM) revealed that lovastatin reduced the number of pseudopodia in MDA-MB-231 but not MDA-MB-453 CSCs (**Figure 5B**). We then examined the effect of lovastatin on cytoskeleton by immunofluorescence-confocal microscopic examination of F-actin. Interestingly, we found F-actin seemed to be changed from diffuse distribution in the cytoplasm in untreated cells to nuclear or perinuclear localization in lovastatin-treated MDA-MB-231 CSCs (**Figure 5C**).

**Figure 6:**
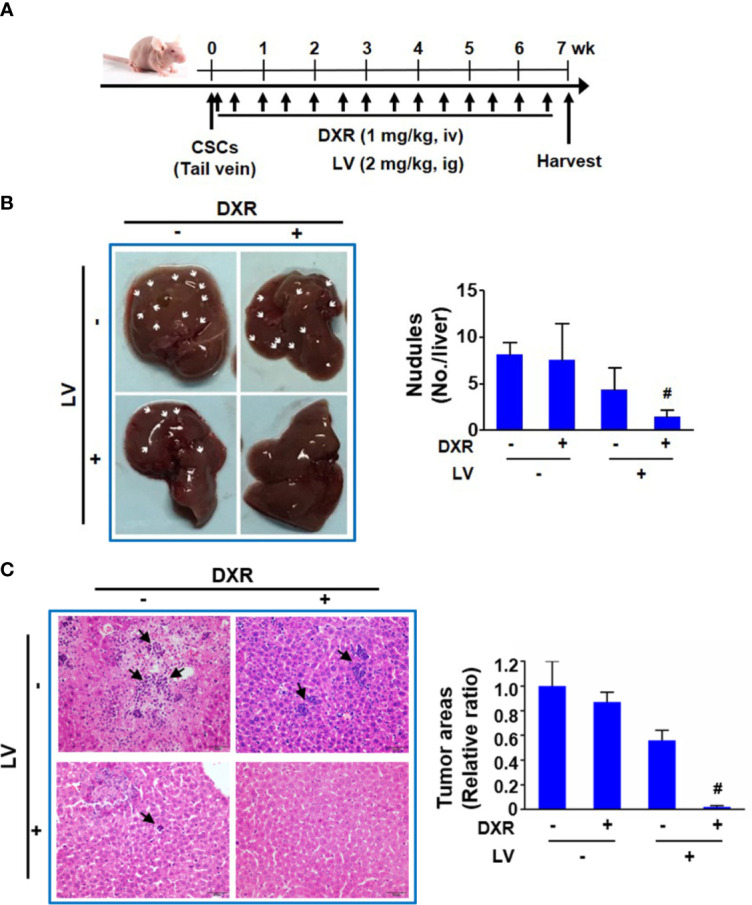
Lovastatin inhibits metastasis of TNBC CSCs *in vivo*. Schematic of the mouse model of tumor metastasis **(A)**. Representative images of the livers from each group of mice. Arrows indicate the tumor nodules on the liver. Right, quantifications of the tumor nodules on liver surface **(B)**. Representative H&E-stained histopathological images showing reduced colonization of tumor cells in the liver by combination treatment with lovastatin and DXR. Arrows indicate the metastatic tumor cells in the liver. Right, quantifications of the metastatic tumor cells on liver surface. Original magnification: 40× **(C)**. ^#^ *CI* < 1.0, showing synergism between the two drugs. LV, lovastatin; DXR, doxorubicin; iv, intravenous injection; ig, intragastric administration.

Another nude mouse model of tail vein injection (**Figure 6A**) was further used to evaluate the synergistic effect of combination treatment on metastasis of TNBC CSCs to distal organs. We found that doxorubicin alone had no inhibition and lovastatin alone showed 46.2 ± 21.7% inhibition on liver metastasis of MDA-MB-231 CSCs. However, combination of lovastatin with doxorubicin synergistically inhibited the majority of liver metastasis of MDA-MB-231 CSCs as demonstrated by a 81.5 ± 5.8% reduction of the macroscopic nodules (**Figure 6B**). Quantification of histopathological examination confirmed the synergistically inhibitory effect on cancer cell colonization in the liver of the combination treatment group (**Figure 6C**). Thus, these data suggest that lovastatin could cause disruption of the cytoskeleton and inhibit liver metastasis in TNBC CSCs.

### Higher Expression Levels of Cytoskeleton-Associated Genes Are Characteristic of TNBC and Predict Survival Outcomes in Breast Cancer Patients

**Figure 7:**
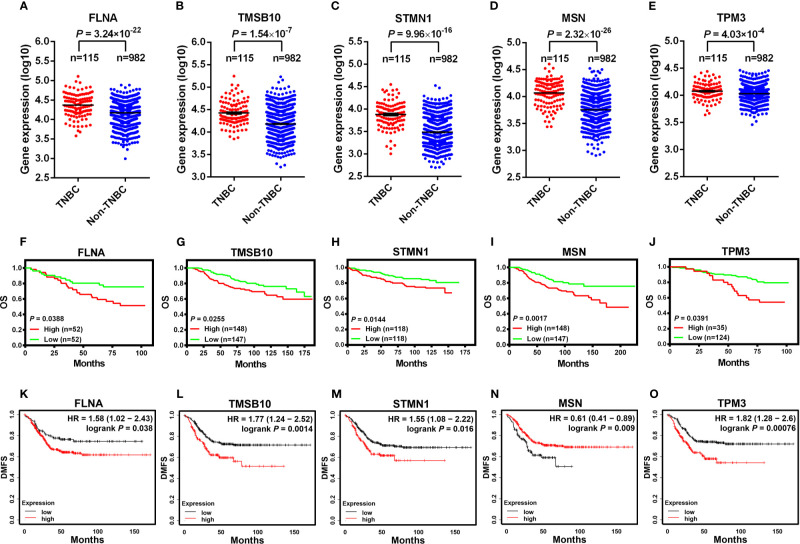
Higher expression levels of cytoskeleton-associated genes are characteristic of TNBC and predict poorer survival outcomes in breast cancer patients. The expression of FLNA **(A)**, TMSB10 **(B)**, STMN1 **(C)**, MSN **(D)**, SPTAN1 **(E)** analyzed between TNBC and non-TNBC clinical samples from The Cancer Genome Atlas (TCGA), which was contained in the online cBioPortal for cancer genomics database **(A-E)**. OS in breast cancer patients retrieved from the online database (PROGgeneV2) between high and low expression of FLNA [**(F)**, dataset GSE42568], TMSB10 [**(G)**, dataset NKI], STMN1 [**(H)**, dataset GSE3494_U133A], MSN [**(I)**, dataset NKI], TPM3 [**(J)**, dataset GSE1456-GPL97] **(F–J)**. DMFS in TNBC patients between high and low expression of cytoskeleton-associated genes on the Kaplan-Meier plotter database **(K-O)**. OS, overall survival; DMFS, distant metastasis-free survival.

In order to explore how our results might be relevant to the clinic, we compared the expression levels of cytoskeleton-related genes between TNBC and non-TNBC and investigated their associations with breast cancer patient survival. We analyzed the RNA-seq data of TNBC and non-TNBC samples from The Cancer Genome Atlas (TCGA) database for the cytoskeleton-related proteins revealed in **Figure 1B**. We found that five out of the eight cytoskeleton-related genes, i.e., FLNA, TMSB10, STMN1, MSN, and TPM3, were expressed at significantly higher levels in TNBC compared with non-TNBC (**Figures 7A–E**). Consistent with the RNA-seq data, survival analysis using the PROGgeneV2 tool showed that breast cancer patients of all molecular subtypes who had higher levels of these cytoskeleton-related genes had poorer OS compared with those with lower levels (**Figures 7F–J**). We further explored the roles of these cytoskeleton-related genes in the survival of TNBC patients using the Kaplan-Meier plotter database. Our results revealed that the expression levels of these genes were associated with distant metastasis-free survival (DMFS) (**Figures 7K–O**) of TNBC patients. Except for MSN, the expression levels of four out of the five genes, i.e., FLNA, TMSB10, STMN1, and TPM3, were negatively associated with DMFS of TNBC patients. The clinical data suggest that these cytoskeleton-related genes might be potential targets for the treatment of TNBC.

## Discussion

It is well known that EMT and metastasis play an important role in the acquisition of the malignant phenotype of cancer cells (41). We set out to explore whether lovastatin could inhibit breast CSCs by reversal of the EMT program and inhibition of metastasis in TNBC. Our study demonstrated that both the expression of mesenchymal markers such as Vimentin and EMT-related transcription factors such as Twist could be down-regulated by treatment with lovastatin in MDA-MB-231 CSCs. Furthermore, EMT induced by TGF-β1 in immortalized mammary epithelial cells MCF10A could also be reversed by lovastatin. The present study also showed that lovastatin could inhibit liver metastasis as evidenced by reduced nodule formation and confirmed histopathologically by eliminated cancer cell colonization. In our animal model, we also examined the lungs for metastasis. Unexpectedly, we didn’t observe metastatic cancer cells in the lungs as obviously did in the liver. One speculation is that the cells we used were CSCs, rather than bulk tumor cells, and the difference in signaling pathways between them may lead to the difference in metastatic spreading of tumor cells to different target organs. These results provided solid evidence that lovastatin could inhibit the EMT program and metastasis in TNBC CSCs *in vitro* and *in vivo*.

In the process of EMT, tumor cells gain migratory and metastatic properties that involve a dramatic reorganization of the actin cytoskeleton and the concomitant formation of membrane protrusions required for invasive growth (41, 42). Emerging evidence suggests that cytoskeleton regulatory proteins are a convergent node of signaling pathways emanating from extracellular stimulus to cell movement. The coordinated activity of various cytoskeleton-binding proteins regulates a variety of cytoskeleton-based processes, including assembly of the microfilament and cell motility.

Actin cytoskeleton remodeling is an upstream regulator of EMT in metastatic breast cancer cells (43), and several studies clarified EMT was driven by actin cytoskeleton remodeling in hepatocellular and colorectal carcinoma (44, 45). F-actin cytoskeleton is regulated by various actin-binding proteins, one family of which are the filamins, with filamin A (FLNA), also called actin-binding protein 280 (ABP-280), being the most powerful actin-binding protein, together with actin microfilaments, direct the cell’s elasticity and movement (46, 47). In our study, we demonstrated lovastatin induced rearrangement of the actin cytoskeleton favoring perinuclear and nuclear localization of F-actin filaments. Location of actin filaments underneath the plasma membrane is important for the formation of cellular protrusions such as lamellipodia and filopodia (48). Our results further showed that the number of pseudopodia of TNBC CSCs after lovastatin-treated were reduced, which confirmed cytoskeleton organization pathway play an important role in the lovastatin inhibition EMT and metastasis of TNBC CSCs. We have demonstrated that lovastatin inhibited the EMT and metastasis of TNBC CSCs. Therefore, it’s not surprising that lovastatin modulates these malignant behaviors of TNBC CSCs through dysregulation of cytoskeleton-associated proteins. This is supported by bioinformatics analysis showing that the cytoskeleton-associated genes are differentially expressed between TNBC and non-TNBC tissues samples and that higher expression levels of these genes are associated with survival outcomes in TNBC patients.

In summary, our present study has provided evidence, for the first time, that lovastatin, a natural HMG-CoA reductase inhibitor, inhibits TNBC CSCs *in vitro* and *in vivo* through inhibition of EMT phenotype and suppression of metastasis by dysregulation of cytoskeleton-associated proteins. This study lays the foundation for the understanding of the inhibitory effect of lovastatin on the EMT and metastasis of TNBC CSCs and has potential clinical implications for the future management of TNBC. Further studies are required to move forward our effort toward resolving the issues of how lovastatin causes disturbance of the cytoskeleton organization pathway and how protein Ksucc contributes to lovastatin-induced EMT and metastasis in TNBC CSCs.

## Data Availability Statement

Publicly available datasets were analyzed in this study. This data can be found here: http://www.cbioportal.org/study/summary?id=brca_tcga.

## Ethics Statement

The animal study was reviewed and approved by Hunan Normal University Institutional Animal Care and Ethics Committee.

## Author Contributions

CZ, SY, LL, HY, and GH performed the experiments. SC, YL, XP, and ZC collected and analyzed the data. CZ, SY, and LL drafted the manuscript. MW, QZ, GL, and SF reviewed the manuscript. XD conceived and designed the research. All authors contributed to the article and approved the submitted version.

## Funding

This work was supported by the National Natural Science Foundation of China (81872167, 81472496), Key Grant of Research and Development in Hunan Province (2020DK2002), Natural Science Foundation of Hunan (2019JJ40193), and Key Project of Department of Education of Hunan Province (14A089).

## Conflict of Interest

XP and ZC were employed by Jingjie PTM BioLab Co. Ltd.

The remaining authors declare that the research was conducted in the absence of any commercial or financial relationships that could be construed as a potential conflict of interest.

## References

[1] Siegel RL Miller KD Jemal A. Cancer Statistics, 2020. CA Cancer J Clin (2020) 70(1):7–30. 10.3322/caac.21590 31912902

[2] Sorlie T Perou CM Tibshirani R Aas T Geisler S Johnsen H. Gene Expression Patterns of Breast Carcinomas Distinguish Tumor Subclasses With Clinical Implications. Proc Natl Acad Sci USA (2001) 98(19):10869–74. 10.1073/pnas.191367098 PMC58566 11553815

[3] Dent R Trudeau M Pritchard KI Hanna WM Kahn HK Sawka CA. Triple-Negative Breast Cancer: Clinical Features and Patterns of Recurrence. Clin Cancer Res (2007) 13(15 Pt 1):4429–34. 10.1158/1078-0432.CCR-06-3045 17671126

[4] Fremd C Jaeger D Schneeweiss A. Targeted and Immuno-Biology Driven Treatment Strategies for Triple-Negative Breast Cancer: Current Knowledge and Future Perspectives. Expert Rev Anticancer Ther (2019) 19(1):29–42. 10.1080/14737140.2019.1537785 30351981

[5] Lee A Djamgoz MBA. Triple Negative Breast Cancer: Emerging Therapeutic Modalities and Novel Combination Therapies. Cancer Treat Rev (2018) 62:110–22. 10.1016/j.ctrv.2017.11.003 29202431

[6] Papadimitriou M Mountzios G Papadimitriou CA. The Role of PARP Inhibition in Triple-Negative Breast Cancer: Unraveling the Wide Spectrum of Synthetic Lethality. Cancer Treat Rev (2018) 67:34–44. 10.1016/j.ctrv.2018.04.010 29753961

[7] Emens LA Cruz C Eder JP Braiteh F Chung C Tolaney SM. Long-Term Clinical Outcomes and Biomarker Analyses of Atezolizumab Therapy for Patients With Metastatic Triple-Negative Breast Cancer: A Phase 1 Study. JAMA Oncol (2019) 5(1):74–82. 10.1001/jamaoncol.2018.4224 30242306 PMC6439773

[8] Liu S Wicha MS. Targeting Breast Cancer Stem Cells. J Clin Oncol (2010) 28(25):4006–12. 10.1200/JCO.2009.27.5388 PMC4872314 20498387

[9] Islam F Qiao B Smith RA Gopalan V Lam AK. Cancer Stem Cell: Fundamental Experimental Pathological Concepts and Updates. Exp Mol Pathol (2015) 98(2):184–91. 10.1016/j.yexmp.2015.02.002 25659759

[10] O’Conor CJ Chen T Gonzalez I Cao D Peng Y. Cancer Stem Cells in Triple-Negative Breast Cancer: A Potential Target and Prognostic Marker. Biomark Med (2018) 12(7):813–20. 10.2217/bmm-2017-0398 29902924

[11] Dey P Rathod M De A. Targeting Stem Cells in the Realm of Drug-Resistant Breast Cancer. Breast Cancer (Dove Med Press) (2019) 11:115–35. 10.2147/BCTT.S189224 PMC6410754 30881110

[12] Ishiwata T. Cancer Stem Cells and Epithelial-Mesenchymal Transition: Novel Therapeutic Targets for Cancer. Pathol Int (2016) 66(11):601–8. 10.1111/pin.12447 27510923

[13] Sato R Semba T Saya H Arima Y. Concise Review: Stem Cells and Epithelial-Mesenchymal Transition in Cancer: Biological Implications and Therapeutic Targets. Stem Cells (2016) 34(8):1997–2007. 10.1002/stem.2406 27251010

[14] Voulgari A Pintzas A. Epithelial-Mesenchymal Transition in Cancer Metastasis: Mechanisms, Markers and Strategies to Overcome Drug Resistance in the Clinic. Biochim Biophys Acta (2009) 1796(2):75–90. 10.1016/j.bbcan.2009.03.002 19306912

[15] Kraljevic Pavelic S Sedic M Bosnjak H Spaventi S Pavelic K. Metastasis: New Perspectives on an Old Problem. Mol Cancer (2011) 10:22. 10.1186/1476-4598-10-22 21342498 PMC3052211

[16] May CD Sphyris N Evans KW Werden SJ Guo W Mani SA. Epithelial-Mesenchymal Transition and Cancer Stem Cells: A Dangerously Dynamic Duo in Breast Cancer Progression. Breast Cancer Res (2011) 13(1):202. 10.1186/bcr2789 21392411 PMC3109556

[17] Zhang Z Tan M Xie Z Dai L Chen Y Zhao Y. Identification of Lysine Succinylation as a New Post-Translational Modification. Nat Chem Biol (2011) 7(1):58–63. 10.1038/nchembio.495 21151122 PMC3065206

[18] Wang C Zhang C Li X Shen J Xu Y Shi H. CPT1A-Mediated Succinylation of S100A10 Increases Human Gastric Cancer Invasion. J Cell Mol Med (2019) 23(1):293–305. 10.1111/jcmm.13920 30394687 PMC6307794

[19] Song Y Wang J Cheng Z Gao P Sun J Chen X. Quantitative Global Proteome and Lysine Succinylome Analyses Provide Insights Into Metabolic Regulation and Lymph Node Metastasis in Gastric Cancer. Sci Rep (2017) 7:42053. 10.1038/srep42053 28165029 PMC5292683

[20] Gao X Bao H Liu L Zhu W Zhang L Yue L. Systematic Analysis of Lysine Acetylome and Succinylome Reveals the Correlation Between Modification of H2A.X Complexes and DNA Damage Response in Breast Cancer. Oncol Rep (2020) 43(6):1819–30. 10.3892/or.2020.7554 PMC7160542 32236595

[21] Chen XF Tian MX Sun RQ Zhang ML Zhou LS Jin L. SIRT5 Inhibits Peroxisomal ACOX1 to Prevent Oxidative Damage and Is Downregulated in Liver Cancer. EMBO Rep (2018) 19(5). 10.15252/embr.201745124 PMC5934778 29491006

[22] Yang X Wang Z Li X Liu B Liu M Liu L. SHMT2 Desuccinylation by SIRT5 Drives Cancer Cell Proliferation. Cancer Res (2018) 78(2):372–86. 10.1158/0008-5472.Can-17-1912 29180469

[23] Li QQ Hao J-J Zhang Z Krane LS Hammerich KH Sanford T. Proteomic Analysis of Proteome and Histone Post-Translational Modifications in Heat Shock Protein 90 Inhibition-Mediated Bladder Cancer Therapeutics. Sci Rep (2017) 7(1):201. 10.1038/s41598-017-00143-6 28298630 PMC5427839

[24] Shi YC Pan TM. Beneficial Effects of Monascus Purpureus NTU 568-Fermented Products: A Review. Appl Microbiol Biotechnol (2011) 90(4):1207–17. 10.1007/s00253-011-3202-x 21455594

[25] Le NA. Hyperlipidaemia and Cardiovascular Disease. Curr Opin Lipidol (2001) 12(5):587–9. 10.1097/00041433-200110000-00016 11561179

[26] Graaf MR Richel DJ van Noorden CJ Guchelaar HJ. Effects of Statins and Farnesyltransferase Inhibitors on the Development and Progression of Cancer. Cancer Treat Rev (2004) 30(7):609–41. 10.1016/j.ctrv.2004.06.010 15531395

[27] Yu X Luo Y Zhou Y Zhang Q Wang J Wei N. BRCA1 Overexpression Sensitizes Cancer Cells to Lovastatin Via Regulation of Cyclin D1-CDK4-p21WAF1/CIP1 Pathway: Analyses Using a Breast Cancer Cell Line and Tumoral Xenograft Model. Int J Oncol (2008) 33(3):555–63. 18695886

[28] Campbell MJ Esserman LJ Zhou Y Shoemaker M Lobo M Borman E. Breast Cancer Growth Prevention by Statins. Cancer Res (2006) 66(17):8707–14. 10.1158/0008-5472.CAN-05-4061 16951186

[29] Kang S Kim ES Moon A. Simvastatin and Lovastatin Inhibit Breast Cell Invasion Induced by H-Ras. Oncol Rep (2009) 21(5):1317–22. 10.3892/or_00000357 19360310

[30] Song L Tao X Lin L Chen C Yao H He G. Cerasomal Lovastatin Nanohybrids for Efficient Inhibition of Triple-Negative Breast Cancer Stem Cells to Improve Therapeutic Efficacy. ACS Appl Mater Interfaces (2018) 10(8):7022–30. 10.1021/acsami.8b01633 29405062

[31] Yang T Yao H He G Song L Liu N Wang Y. Effects of Lovastatin on MDA-MB-231 Breast Cancer Cells: An Antibody Microarray Analysis. J Cancer (2016) 7(2):192–9. 10.7150/jca.13414 PMC4716852 26819643

[32] Yao H He G Yan S Chen C Song L Rosol TJ. Triple-Negative Breast Cancer: Is There a Treatment on the Horizon? Oncotarget (2017) 8(1):1913–24. 10.18632/oncotarget.12284 PMC5352107 27765921

[33] Li Y Zhang T Korkaya H Liu S Lee HF Newman B. Sulforaphane, a Dietary Component of Broccoli/Broccoli Sprouts, Inhibits Breast Cancer Stem Cells. Clin Cancer Res (2010) 16(9):2580–90. 10.1158/1078-0432.CCR-09-2937 PMC2862133 20388854

[34] Peng Y He G Tang D Xiong L Wen Y Miao X. Lovastatin Inhibits Cancer Stem Cells and Sensitizes to Chemo- and Photodynamic Therapy in Nasopharyngeal Carcinoma. J Cancer (2017) 8(9):1655–64. 10.7150/jca.19100 PMC5535721 28775785

[35] Chiu HW Fang WH Chen YL Wu MD Yuan GF Ho SY. Monascuspiloin Enhances the Radiation Sensitivity of Human Prostate Cancer Cells by Stimulating Endoplasmic Reticulum Stress and Inducing Autophagy. PloS One (2012) 7(7):e40462. 10.1371/journal.pone.0040462 22802963 PMC3389026

[36] Zhang J Zhang X Li Z Wang Q Shi Y Jiang X. The Mir-124-3p/Neuropilin-1 Axis Contributes to the Proliferation and Metastasis of Triple-Negative Breast Cancer Cells and Co-Activates the TGF-β Pathway. Front Oncol (2021) 11:654672. 10.3389/fonc.2021.654672 33912463 PMC8072051

[37] Li H He G Yao H Song L Zeng L Peng X. TGF-Beta Induces Degradation of PTHrP Through Ubiquitin-Proteasome System in Hepatocellular Carcinoma. J Cancer (2015) 6(6):511–8. 10.7150/jca.10830 PMC4439935 26000041

[38] Lee S. Post-Translational Modification of Proteins in Toxicological Research: Focus on Lysine Acylation. Toxicol Res (2013) 29(2):81–6. 10.5487/tr.2013.29.2.081 PMC3834447 24278632

[39] Zhang J Tian XJ Zhang H Teng Y Li R Bai F. TGF-Beta-Induced Epithelial-to-Mesenchymal Transition Proceeds Through Stepwise Activation of Multiple Feedback Loops. Sci Signal (2014) 7(345):ra91. 10.1126/scisignal.2005304 25270257

[40] Sotiropoulou PA Christodoulou MS Silvani A Herold-Mende C Passarella D. Chemical Approaches to Targeting Drug Resistance in Cancer Stem Cells. Drug Discovery Today (2014) 19(10):1547–62. 10.1016/j.drudis.2014.05.002 24819719

[41] Yilmaz M Christofori G. EMT, the Cytoskeleton, and Cancer Cell Invasion. Cancer Metastasis Rev (2009) 28(1-2):15–33. 10.1007/s10555-008-9169-0 19169796

[42] Kalluri R Weinberg RA. The Basics of Epithelial-Mesenchymal Transition. J Clin Invest (2009) 119(6):1420–8. 10.1172/JCI39104 PMC2689101 19487818

[43] Shankar J Nabi IR. Actin Cytoskeleton Regulation of Epithelial Mesenchymal Transition in Metastatic Cancer Cells. PloS One (2015) 10(3):e0119954. 10.1371/journal.pone.0119954 25756282 PMC4355409

[44] Peng JM Bera R Chiou CY Yu MC Chen TC Chen CW. Actin Cytoskeleton Remodeling Drives Epithelial-Mesenchymal Transition for Hepatoma Invasion and Metastasis in Mice. Hepatology (2018) 67(6):2226–43. 10.1002/hep.29678 29171033

[45] Sousa-Squiavinato ACM Rocha MR Barcellos-de-Souza P de Souza WF Morgado-Diaz JA. Cofilin-1 Signaling Mediates Epithelial-Mesenchymal Transition by Promoting Actin Cytoskeleton Reorganization and Cell-Cell Adhesion Regulation in Colorectal Cancer Cells. Biochim Biophys Acta Mol Cell Res (2019) 1866(3):418–29. 10.1016/j.bbamcr.2018.10.003 30296500

[46] Pollard TD Cooper JA. Actin, a Central Player in Cell Shape and Movement. Science (2009) 326(5957):1208–12. 10.1126/science.1175862 PMC3677050 19965462

[47] Schmoller KM Lieleg O Bausch AR. Structural and Viscoelastic Properties of Actin/Filamin Networks: Cross-Linked Versus Bundled Networks. Biophys J (2009) 97(1):83–9. 10.1016/j.bpj.2009.04.040 PMC2711384 19580746

[48] Maninova M Caslavsky J Vomastek T. The Assembly and Function of Perinuclear Actin Cap in Migrating Cells. Protoplasma (2017) 254(3):1207–18. 10.1007/s00709-017-1077-0 28101692

