# Supplementary material for: Lovastatin Inhibits EMT and Metastasis of Triple-Negative Breast Cancer Stem Cells Through Dysregulation of Cytoskeleton-Associated Proteins
Source: Front Oncol. 2021 Jun 4;11:656687. doi: 10.3389/fonc.2021.656687 (PMC8212055; doi:10.3389/fonc.2021.656687)
Supplement: Supplementary file 1 [file DataSheet_1.docx]

**
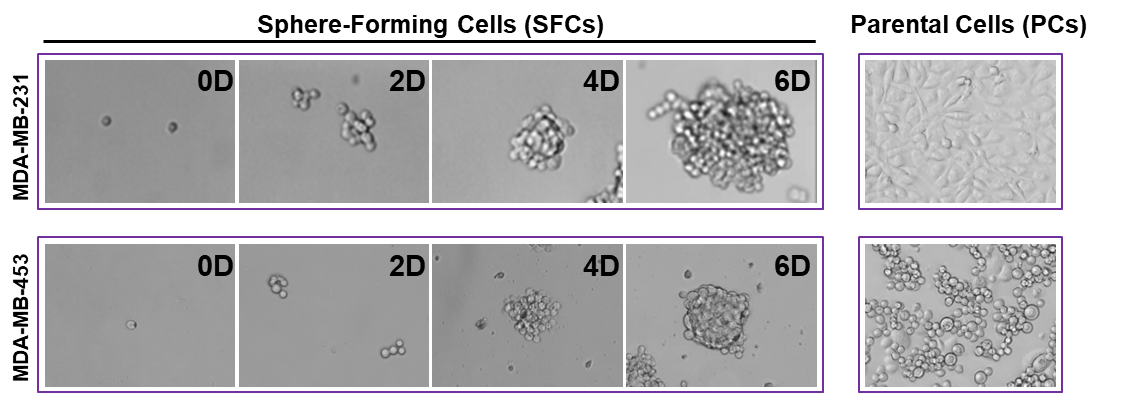
**

**Supplementary Figure 1.** Culture of breast cancer stem-like cells. MDA-MB-231 and MDA-MB-453 sphere-forming cells (SFCs) were enriched from parental cells (PCs) and maintained in stem cell culture medium. These cells were further sorted by magnetic separation for the phenotype of CD44^+^/CD24^-^. The microscopic images were acquired using inverted phase contrast microscope. Original magnification: 200×.

**Supplementary Table 1. Subcutaneous tumor formation in nude mice**

| **Cell Types** | **No. of cells inoculated** | **Tumor incidence** | **Latency period (d)** |
| --- | --- | --- | --- |
| MDA-MB-231-PCs | 1 x 10^4^ | 0/6 | - |
|  | 1 x 10^5^ | 6/6 | 15 |
|  | 1 x 10^6^ | 6/6 | 8 |
| MDA-MB-231-SFCs | 1 x 10^2^ | 0/6 | - |
|  | 1 x 10^3^ | 1/6 | 15 |
|  | 1 x 10^4^ | 6/6 | 7 |

PC, Parental Cells; SFC, Sphere-Forming Cells

**
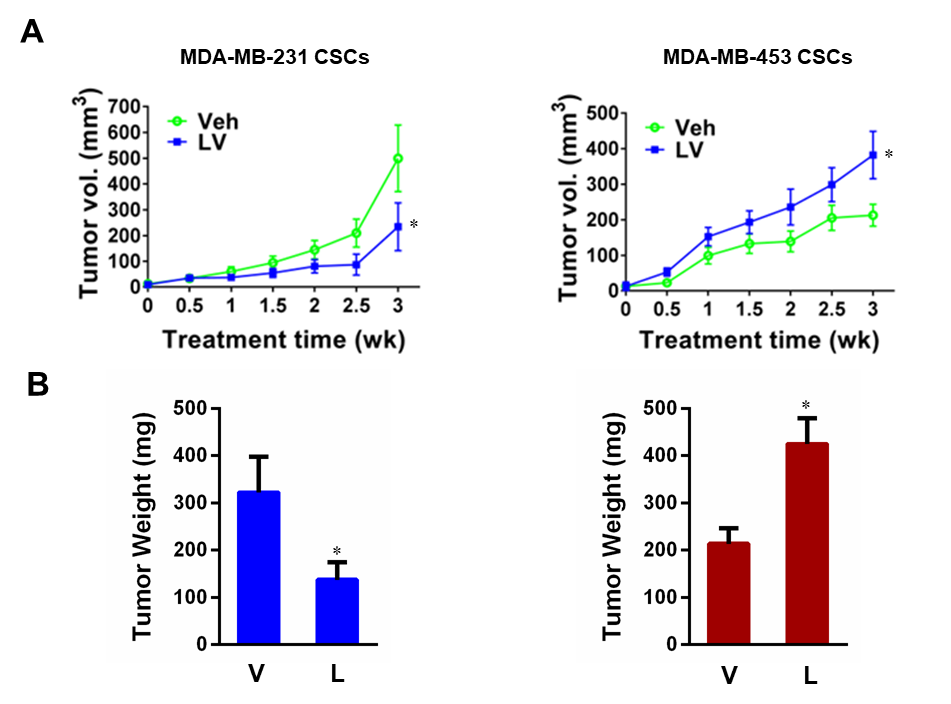
**

**Supplementary Figure 2.** Lovastatin suppresses growth of orthotopic tumors in nude mice derived from MDA-MB-231 CSCs but not MDA-MB-453 CSCs. Change of tumor volume over time in different groups **(A)**. The tumor weight of the mice in different groups. Data are shown as mean ± SEM **(B)**.

* *P* < 0.05, compared with control; V or Veh, vehicle; L or LV, lovastatin.
